# Comparison of the therapeutic effects of sacubitril and valsartan combination versus metformin in experimentally induced polycystic ovary syndrome in rats

**DOI:** 10.22038/ijbms.2025.87940.18996

**Published:** 2025

**Authors:** Muhammed Yayla, Bengul Ozdemir Sarikaya, Erdem Toktay, Huseyin Fatih Gul, Ugur Ermis, Damla Binnetoglu

**Affiliations:** 1 Department of Pharmacology, Faculty of Medicine, Selcuk University, 42100, Konya, Turkey; 2 Department of Histology and Embryology, Faculty of Medicine, Kafkas University, 36100, Kars, Turkey; 3 Department of Medical Biochemistry, Faculty of Medicine, Kafkas University, 36100, Kars, Turkey; 4 Department of Pharmacology, Faculty of Medicine, Kafkas University, 36100, Kars, Turkey

**Keywords:** ARB, Insulin resistance, Neprilysin, PCOS, Rat, Sacubitril, Valsartan

## Abstract

**Objective(s):**

Our study aimed to demonstrate the therapeutic effects of sacubitril (an inhibitor of neprilysin) and/or valsartan (an ARB) in an experimentally induced polycystic ovary syndrome (PCOS) model and in PCOS-induced insulin resistance.

**Materials and Methods:**

After 21 days of letrozole 1 mg/kg administration, rats were confirmed to have PCOS by the vaginal smear method. Following PCOS induction, the experiment was terminated after 15 days of drug treatment. Metformin 300 mg/kg, sacubitril 30 mg/kg, and valsartan 31 mg/kg were administered orally every 15 days. Fasting insulin levels and oral glucose tolerance test (OGTT) were performed to measure insulin resistance and calculate homeostatic model assessment - insulin resistance (HOMA-IR). At the end of the experiment, biochemical analyses were performed on blood samples, and histological studies were conducted on tissue samples.

**Results:**

The sacubitril and valsartan combination significantly improved impaired glucose tolerance and HOMA-IR. Serum neprilysin (NEP) levels were found to be significantly higher in the PCOS group than in the healthy group, while atrial natriuretic peptide (ANP) and brain natriuretic peptide (BNP) levels were found to be significantly lower. The sacubitril+valsartan combination provided the greatest improvement in PCOS-related changes in serum NEP, ANP, BNP, angiotensin II (ANGII), hormone, and lipid levels. The application of sacubitril+valsartan provided an important treatment for insulin resistance due to PCOS by increasing the expression of insulin resistance (IR), insulin receptor substrate 1 (IRS-1), and insulin receptor substrate 2 (IRS-2).

**Conclusion:**

Sacubitril and valsartan combination has been shown to have significant therapeutic benefits for PCOS. It markedly reduces cystic follicles and PCOS-associated insulin resistance, improves serum lipid levels, and is supported by pathological findings.

## Introduction

Polycystic ovary syndrome (PCOS) is a common hormonal disorder affecting women of childbearing age. According to the United States (US) National Institutes of Health, the prevalence is 6-10%. PCOS is an endocrine disorder and a leading cause of infertility (1). In addition, untreated PCOS can be associated with diabetes, heart disease, and metabolic syndrome (2). Common clinical signs include menstrual irregularities and chronic anovulation, which are often manifested as oligomenorrhoea or amenorrhoea. Other signs include polycystic ovaries, hirsutism as revealed by ultrasonography, hyperandrogenism (such as acne and alopecia), and insulin resistance (IR). PCOS typically presents at or just after puberty. The aetiology of PCOS is unknown, but the syndrome is often associated with obesity, IR, and metabolic disorders. 

Neprilysin (also known as neutral endopeptidase or NEP) is a protein found in the plasma membrane of many tissues, including pancreatic islets, endothelial cells, smooth muscle cells, epithelial cells, mesenteric adipose tissue, and fibroblasts (3). The NEP enzyme is responsible for degrading many vasoactive peptides, including atrial natriuretic peptide (ANP), brain natriuretic peptide (BNP), bradykinin, and glucagon-like peptide 1 (GLP-1), as well as angiotensin II (4). NEP is also produced by adipocytes, suggesting that it may be an adipokine involved in regulating adipocyte function. One of the most important roles of neprilysin is to break down essential peptides in the body, particularly ANP and BNP. ANP is also known to regulate ovarian functions, such as follicular growth and steroid hormone production, and its levels are decreased in patients with PCOS (5, 6). Clinical studies have shown that NEP levels increase in women with PCOS, while ANP levels decrease in parallel. This suggests that NEP enzyme activity may play an important role in the pathophysiology of PCOS (7). The fact that NEP enzyme activity contributes to both IR pathophysiology (8, 9) and PCOS pathophysiology (7) suggests that NEP inhibitors may have a potential role in the strategies to be developed for PCOS treatment. There are also studies showing increased renin–angiotensin–aldosterone system (RAAS) activity in PCOS, and it is thought that this increased level may cause excessive androgen production in the condition. Theoretically, RAAS is also believed to increase the risk of diabetes by increasing IR and/or insulin secretion. A study of patients with PCOS found that plasma angiotensin-converting enzyme (ACE) and angiotensin II levels were significantly higher (10).

Sacubitril (a neprilysin inhibitor) and valsartan (an angiotensin receptor blocker) are currently approved in combination for treating heart failure (HF with reduced ejection fraction). Studies have shown that this combination may have significant effects in preventing diabetes and controlling glycaemia (11-13). In this context, demonstrating the efficacy of the sacubitril and valsartan combination for treating PCOS and insulin resistance (IR) due to PCOS aims to reduce the number of medications required for patients with PCOS (and IR) and cardiovascular disease. Therefore, we aimed to compare the therapeutic effects of the neprilysin inhibitor sacubitril and ARB valsartan, both alone and in combination, in the treatment of PCOS and PCOS-related IR.

## Materials and Methods

### Experimental animal

In our study, 60 adult (4-6 weeks old) female Sprague-Dawley rats weighing 160-180 g were used. Animals were fed *ad libitum* with standard laboratory feed pellets and tap water at a temperature of 25±5 ^°^C, with a 12-hour night and 12-hour day cycle. Animals were obtained from Kafkas University Experimental Research and Application Center. This study was approved by and performed in accordance with the institutional animal care and use ethics committee of Kafkas University with the number 2023-039. Solvents of Drugs Used in the Experiment: Metformin, sacubitril, and valsartan were dissolved in distilled water, and letrozole was dissolved in 0.5% carboxymethyl cellulose (CMC) and administered orally. Sacubitril was obtained from Ali Raif Drug Company (Turkey). 

### Animal model and experimental groups

All animals were examined by vaginal cytology for regular estrus for 4 days before the experiment, and animals were divided into six groups. To create a PCOS model, 1 mg/kg letrozole was dissolved in 0.5% CMC and given orally for 21 days to the other five groups except the healthy group (14). From day 21, vaginal cytology was performed for four days to determine whether the PCOS pattern was established, and rats with irregular estrus and glucose intolerance were considered PCOS positive (15). In rats developing PCOS, drug administration was performed for 15 days according to the following groups. Weekly weight monitoring was performed.

### Groups

1. Healthy (0.5% orally CMC).

2. PCOS (1 mg/kg Letrozol)(14)

3. PCOS+Metformin (After PCOS induction, 300 mg/kg) (16)

4. PCOS+Sacubitril (After PCOS induction, 30 mg/kg Sacubitril)(17).

5. PCOS+Valsartan (After PCOS induction, 31 mg/kg valsartan)(18).

6. PCOS+Sacubitril+valsartan (After PCOS induction, 68 mg/kg sacubitril+Valsartan)(18).

Animals were fasted for one night beforehand. All animals were injected intraperitoneally with a mixture of 75 mg/kg ketamine (Ketalar, Pfizer) and 15 mg/kg Xylazine (Xylanzinbio, Bioveta). After anesthesia, intracardiac blood was collected and placed in plain biochemistry tubes for routine biochemical and hormone analyses and in anticoagulated tubes for other peptide analyses. Then ovarian (The ovaries were collected and weighed on a sensitive scale, and their weights were recorded), liver, fat, muscle, and pancreas tissues were also removed. The tissues were placed in 10% formalin solution for histologic examination and at -80 degrees Celsius for biochemical analysis.

### Vaginal smear

**Figure 1:**
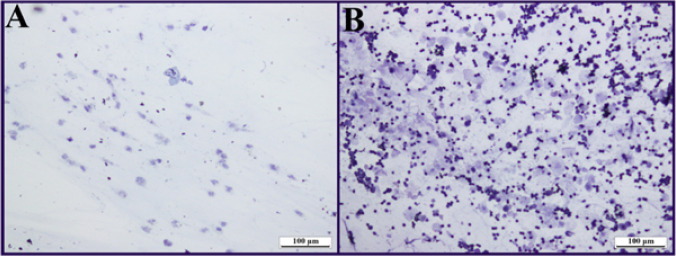
Vaginal smear test results of letrozole induced pcos in rats

The estrus cycle in rats lasts approximately 4-5 days. To determine the stages of the cycle, a moistened swab was inserted into the vagina of the animals and rotated around the vaginal wall to collect smears, spread on slides, air-dried to allow cell detection, stained with Giemsa stain (a rapid method), washed in running water to remove excess stain, and then examined and evaluated under a microscope for cellular structure, including the presence or absence of nuclei, cytoplasm, and neutrophils. While only cornified epithelial cells were observed in the estrus phase in the PCOS-treated groups (Figure 1-A), cornified epithelial cells and leukocytes were observed in the metaestrus phase in the healthy group animals (Figure 1-B).

### Oral glucose tolerance test (OGTT)

It is applied to evaluate insulin release and resistance. At the end of the experiment, all rats were fasted the night before, and after oral administration of 2 g/kg glucose, blood samples were taken from the tail vein at 0, 60, and 120 hr. Glucose levels were measured using a glucose meter (on cal plus)(15)

### Biochemical analyzes

Follicle-stimulating hormone (FSH), Luteinizing hormone (LH), estrogen, testosterone, insulin hormone, Low-density lipoprotein (LDL), High-density lipoprotein (HDL), triglyceride (TG), and total cholesterol (T.COL) levels in serum samples, as well as NEP, Ang-II, ANP, and BNP peptide levels in plasma samples were measured using the Enzyme-Linked ImmunoSorbent Assay (ELISA) method. RAT-ELISA kits were purchased from the Cloud-Clone Corp. (CCC, USA), and absorbance readings were measured on the Epoch™ Microplate Spectrophotometer (BioTek Instruments, USA) using appropriate colorimetric methods as specified in the company procedure.

### Histological analysis

Formalin-fixed ovary, liver, pancreas, muscle, and visceral adipose tissues were blocked after tissue tracing procedures, and 5 µm sections were prepared on positively charged slides for histopathological and immunohistochemical examination in a microtome device (19).

### Hematoxylin-eosin staining protocol

The sections taken from the blocks were deparaffinized in an oven at 60 ^°^C for 30 min, then kept in Xylol-I for 20 min, Xylol-II for 10 min, 100%, 96%, 80%, and 70% alcohols for 2 min each, distilled water for 3 min, and then kept in hematoxylin stain for 10 min and washed in running water for 5 min to stain the nuclei. Then they were stained in Eosin Y solution for 5 min and quickly passed through 70%, 80%, 96%, and 100% alcohols, kept in xylols for 20 and 10 min each, and covered with Entellan (20).

### Immunohistochemical analysis

After 5-μm sections from the blocked, paraffin-embedded tissues were taken on positively charged slides for immunohistochemical analysis, procedures that have been described in previous studies (21) were followed. Antibodies used in staining, IR (PAD895Ra01, Cloud-Clone Corp., Wuhan, China), insulin receptor substrate 1 (IRS-1)(PAC546Mu01, Cloud-Clone Corp., Wuhan, China), insulin receptor substrate 2 (IRS-2)(PAD880Ra01, Cloud-Clone Corp., Wuhan, China), primary antibodies were used according to the recommended dilution ratio (1/200) (Cloud-Clone Corp., Wuhan, China).

### Microscopic examination and photography

After hematoxylin-eosin and immunohistochemical staining, the preparations were examined and photographed using light microscopy (Olympus BX43) and a camera (Olympus DP21). 100x, 200x, and 400x magnifications were used with the microscope. The micrographs were combined for comparative analysis using the Adobe Photoshop CS6 program. Histopathologic examinations were performed double blind. Immunoreactivity was scored as immune negative-(0), mildly positive-(1), moderately positive-(2) and strongly positive-(3).

### Statistical analysis

 The data obtained from our study were first tested for normal distribution. According to the Kolmogorov-Smirnov tests, it was determined that our data fit a normal distribution. Since there were more than two independent groups in our study, a one-way ANOVA test was used to compare variables, and the Tukey test was used as a post hoc test. Mean and standard deviation values of the data were used. *P*<0.05 was considered statistically significant. The SPSS 21.00 program package was used for data analysis.

## Results

### Body weight results

**Figure 2:**
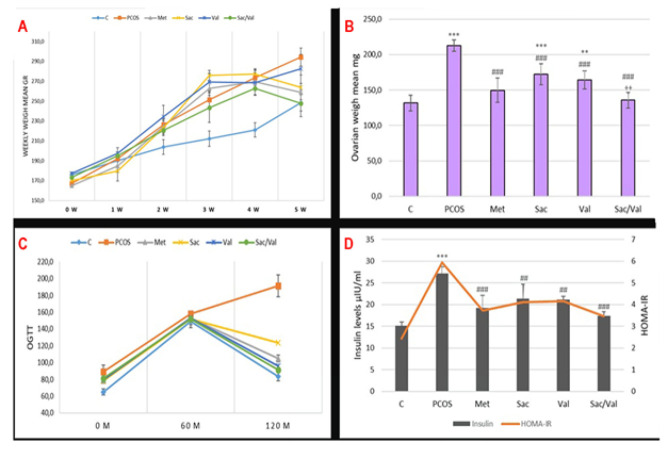
A) Weekly body weight of rats, B) Ovarian weight values, C) OGTT results, D) Fasting insulin levels and HOMA-IR results

As seen in Figure 2A, it is observed that the mean weight of rats with PCOS increased compared to the control group (*P*<0.05). In the Sacubitril+Valsartan and Metformin groups, the mean weights were found to be lower compared to the PCOS group (*P*<0.05). In the groups given sacubitril and valsartan alone, it was observed that the trend of weight gain continued mainly in the valsartan group.

### Overweight results

When Figure 2B is evaluated, it is seen that the mean ovarian weights of the rats that developed PCOS increased compared to the control group (*P*<0.05). In the sacubitril+Valsartan groups, the mean ovarian weights were significantly decreased compared to the PCOS group (*P*<0.05). Mean ovarian weights were also reduced in metformin, sacubitril, and valsartan groups, *P*<0.05.

### Biochemistry results

*OGTT*

Figure 2C presents the results of OGTT. After PCOS-induced IR, the blood glucose level was still high at the 2nd hour in the PCOS group (*P*<0.05). When compared with the control group, it is observed that blood glucose levels decreased at the 2nd hour after OGTT. This situation indicates the development of IR in rats with PCOS. Sacubitril+Valsartan combination administration significantly lowered blood glucose at the end of the 2nd hour in OGTT measurements (*P*<0.05). In the metformin, sacubitril, and valsartan groups, blood glucose levels decreased at the 2nd hour compared to the PCOS group (*P*<0.05). 

### Fasting insulin level

When Figure 2D is analyzed, it is seen that the blood insulin levels of rats with PCOS are significantly higher than those of the control rats. In addition to increased insulin levels, impaired OGTT results are an essential indicator of IR development. It was observed that insulin levels in the sacubitril, valsartan, and combination groups approached normal compared to the PCOS group (*P*<0.05). The best improvement was observed in the combination group and the metformin group.

### Homeostatic model assessment-insulin resistance (HOMA-IR)

When the HOMA-IR findings in Figure 2D are evaluated, it is seen that the HOMA-IR value increased significantly in the PCOS group compared to the control group (*P*<0.05). This calculation shows IR resistance development in rats with PCOS. There was a significant improvement in the HOMA-IR value in the sacubitril, valsartan, and combination groups compared to the PCOS group (*P*<0.05). The best improvement was observed in the sacubitril+valsartan combination and the metformin group.

### Serum hormone results

**Figure 3:**
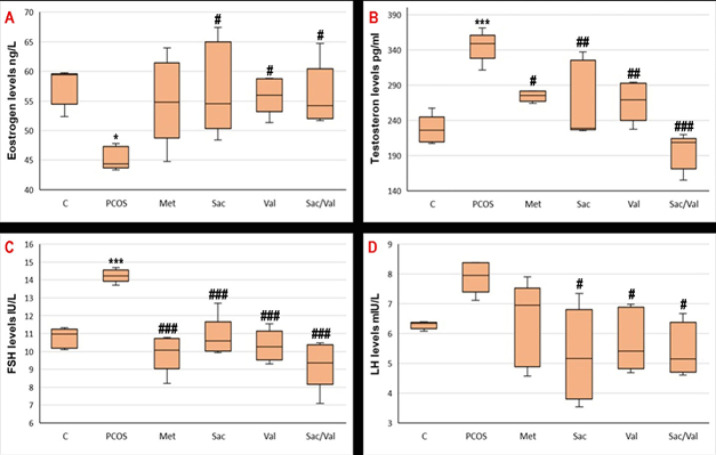
Serum hormon concentrations in Letrozole induced PCOS model

Figure 3A-D shows the difference between the blood hormone levels of PCOS-induced rats. It is observed that estrogen level decreased (*P*<0.05), testosterone level increased (*P*>0.05), and LH (*P*>0.05) and FSH levels increased statistically significantly (*P*<0.05) in rats that developed PCOS. When hormone levels were compared after treatment, it was noted that the sacubitril, valsartan, and combination groups showed a significant improvement in blood hormone levels compared to the PCOS group (*P*<0.05). Increased testosterone levels in the PCOS group improved better in the sacubitril+valsartan combination group.

### Plasma NEP, ANP, BNP, and AngII results

**Figure 4:**
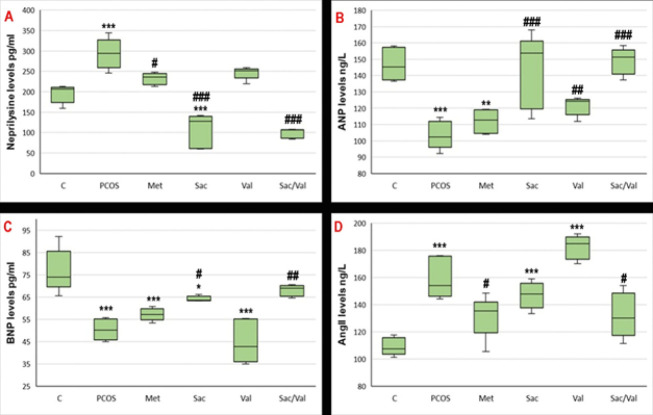
Plasma vasoactive peptides level of letrozole induced PCOS model

When Figure 4A-D is examined, it is observed that the NEP level in the PCOS group increased significantly compared to the control group (*P*<0.05). In the group given NEP inhibitor sacubitril, NEP levels decreased significantly in both the PCOS and control groups (*P*<0.05). Furthermore, when sacubitril was combined with valsartan, NEP levels decreased significantly compared to both the PCOS and control groups (*P*<0.05). In the metformin and valsartan groups, NEP levels decreased compared to the PCOS group, but remained numerically higher than those in the control group. Figure 2E-H shows that levels of ANP and BNP were significantly reduced in the PCOS group, while AngII levels increased, due to the rise in NEP levels. After administering the NEP inhibitor sacubitril, ANP and BNP levels increased significantly compared to the PCOS group (*P*<0.05). Similarly, ANP and BNP levels increased significantly in the group that received a combination of valsartan and sacubitril (*P*<0.05). When analyzing AngII levels, an increase was observed in the PCOS group compared to the control (*P*<0.05). In the Valsartan group, AngII levels increased numerically compared to the PCOS group. A significant decrease was noted in the sacubitril and Valsartan group compared to the PCOS group (*P*<0.05). 

### Serum lipid level

**Figure 5:**
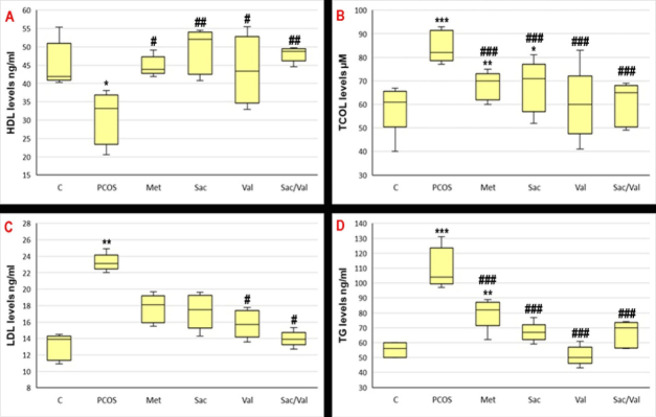
Serum lipid levels of level of letrozole induced PCOS model

When Figure 5A-D is examined, a significant increase was observed in serum LDL, total cholesterol, and TG levels of rats with PCOS compared to the control. In contrast, a significant decrease was observed in HDL level (*P*<0.05). While significant improvement was observed in serum LDL levels in all treatment groups, the best improvement was observed in the Sacubitril and Valsartan groups (*P*<0.05). When serum TG levels were analyzed, a significant improvement was observed in all groups, and the best improvement was observed in the Valsartan group (*P*<0.05). Although there was a numerical decrease in serum total cholesterol levels in all groups, a significant improvement was observed only in the valsartan and sacubitril+valsartan combination groups (*P*<0.05). 

### Histology results

*Hematoxylin and eosin staining results*

**Figure 6:**
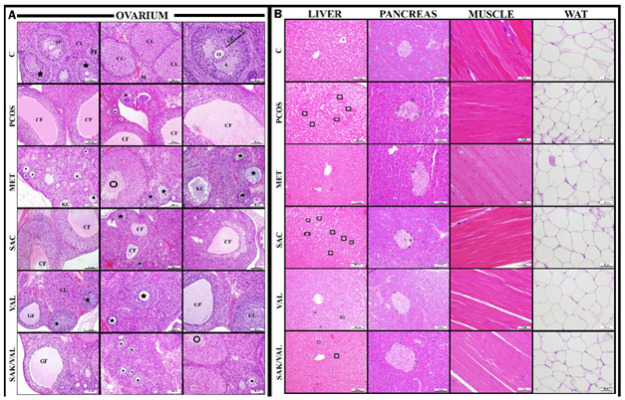
A) Hematoxylin and Eosin (H&E) staining findings in ovarian tissue B) Hematoxylin and Eosin (H&E) staining findings in liver, pancreas, muscle, and adipose tissues

In the histopathologic evaluation of the ovarian tissue, large cystic follicles with antral fluid were observed in the cortex in the PCOS group (Figure 6A). In the metformin, valsartan, and sacubitril+valsartan groups, cystic follicles were rarely seen in the ovary, and the size of the rare cystic follicles was significantly reduced. In the sacubitril group, numerous follicular and corpus luteum cysts were observed, as seen in the PCOS group. 

In the liver tissue, steatosis was observed in the PCOS group as lipid deposits around the hepatocyte cells (Figure 6B). In the metformin group, no lipid accumulations were observed in the liver tissue, whereas in the sacubitril group, steatosis was observed. In the valsartan and sacubitril+valsartan groups, the general appearance was similar to that of metformin and healthy groups, but lipid deposits were observed rarely. 

In the pancreatic tissue, cells with unstained cytoplasm were observed in the peripheral parts of the islands of Langerhans, forming the endocrine pancreas in PCOS and sacubitril groups (Figure 6B). These cells with unstained cytoplasm were rarely observed in the valsartan and sacubitril+valsartan groups. 

In the histopathologic evaluation of muscle and adipose tissues, no pathologic findings were found between the groups (Figure 5B).

### Immunohistochemical staining results

**Figure 7:**
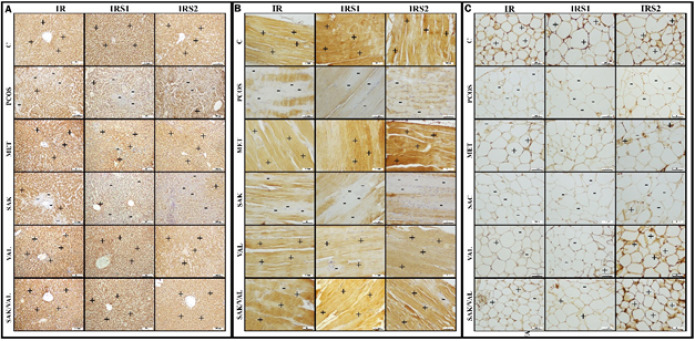
A) Immunohistochemical staining findings of IR, IRS1, and IRS2 in liver tissue of rats, B) Immunohistochemical staining findings of IR, IRS1, and IRS2 in muscle tissue of rats, C) Immunohistochemical staining findings of IR, IRS1, and IRS2 in adipose tissue of rats

Immunohistochemical staining of liver tissue with IR antibody showed mild immunopositivity in the PCOS group, while it was severe in the metformin, valsartan, and sacubitril+valsartan groups (Figure 7A and Table 1). IRS1 antibody revealed mild immunopositivity in the PCOS group and severe immunopositivity in the sacubitril, valsartan, and sacubitril+valsartan groups. IRS2 antibody showed mild immunopositivity in the PCOS group, moderate immunopositivity in the sacubitril+valsartan group, and severe immunopositivity in the sacubitril, valsartan, and metformin groups.

Immunohistochemical staining of muscle tissue with IR antibody showed moderate immunopositivity in the PCOS, sacubitril, and sacubitril+valsartan groups and severe immunopositivity in the valsartan and metformin groups (Figure 7B and Table 1). IRS1 antibody showed mild immune positivity in the PCOS and sacubitril groups, and severe immune positivity in the metformin and sacubitril+valsartan groups. IRS2 antibody showed mild immunopositivity in the PCOS and sacubitril groups, moderate immunopositivity in the sacubitril+valsartan group, and severe immunopositivity in the metformin and valsartan groups.

Immunohistochemical staining of adipose tissue with IR antibody showed mild immunopositivity in PCOS, sacubitril, and valsartan groups; moderate immunopositivity in metformin and sacubitril+valsartan groups (Figure 7C and Table 1). IRS1 antibody showed mild immunopositivity in PCOS, sacubitril, and valsartan groups; moderate immunopositivity in metformin and sacubitril+valsartan groups. IRS2 antibody showed mild immunopositivity in PCOS, sacubitril, and severe immunopositivity in C, valsartan, and sacubitril+valsartan groups.

## Discussion

In letrozole-induced PCOS, there is a decrease in estrogen levels and an increase in testosterone levels, primarily due to the inhibition of peripheral aromatase. Consequently, FSH and LH levels rise, disrupting the estrous cycle in animals with PCOS. While hormonal levels are essential for diagnosing PCOS, the gold standard is a radiological or histological demonstration of cystic follicles in the ovaries (22). Since cystic follicles are larger and contain fluid, they also cause a visible increase in ovarian volume and weight. Ragy *et al*. showed that in their PCOS model induced by letrozole, serum estrogen levels decreased, testosterone levels increased, cystic follicles developed, and lipid levels rose. They also demonstrated that administering hemin and L-arginine increased estrogen and testosterone levels, reduced cystic follicles, and lowered lipid levels due to the overall improvement (23). Zavare *et al*. experimentally induced PCOS with letrozole and showed decreased estrogen levels, increased testosterone levels, and the development of cystic follicles. They also observed that tetrahydrocannabinol-9 treatment improved cystic follicles (24). Our study confirmed that, similar to previous research, serum estrogen decreased, testosterone increased, cystic follicles formed, and ovarian weights grew in letrozole-treated groups. Blood hormone improvements (oestrogen, testosterone, FSH, LH) were notable in treatment groups. The sacubitril+valsartan group showed the most testosterone increase. Histology showed no significant cyst reduction with sacubitril alone, but significant decreases in metformin, valsartan, and the combination group. Ovarian weights in the sacubitril+valsartan group resembled those of the controls. These results suggest sacubitril and valsartan together may be an effective PCOS treatment.

In our study, the NEP level increased, and the ANP and BNP levels decreased in rats with PCOS, while the AngII level increased. In the sacubitril group, the NEP level decreased, and the ANP and BNP levels increased, as did the AngII level. In the valsartan group, there was no significant decrease in NEP levels, while ANP and AngII levels increased, and BNP levels decreased. In the sacubitril and valsartan group, the NEP level decreased, while the ANP and BNP levels increased, and the AngII level decreased. Combining the two drugs in the treatment strategy led to a more significant improvement in ANP, BNP, and AngII levels. Previous studies have shown that NEP levels increase in women who develop PCOS, while ANP and BNP levels decrease (2, 7). Increased NEP activity or decreased ANP and BNP levels contribute to the pathophysiology of PCOS. Pereira *et al*. noted that NEP activity increased and ANP levels decreased in the PCOS model induced by estrogen. In this study, they stated that low ANP levels may indicate an abnormal imbalance in steroid hormones and that this condition may contribute to the development of PCOS (6). Moro *et al*. showed that ANP levels are high in obese women with PCOS and that ANP levels decrease with lipolysis associated with aerobic exercise (21). Also, Zheng *et al.* demonstrated the therapeutic effect of ANP administration in an experimental PCOS model (22). In addition, studies are showing that ACE polymorphism develops and AngII levels increase in women who develop PCOS (23). Because increased ACE activity and AngII levels have been shown to contribute to the pathophysiology of PCOS (23). Sacubitril+valsartan, a combination of a neprilysin inhibitor and an angiotensin II receptor blocker, is approved for HF with reduced EF (24, 25). These drugs increase natriuretic peptide levels by inhibition of Neprilysin (26). Induces diuresis, natriuresis, and extracellular volume reduction, also potentiates the effects of renin-angiotensin-aldosterone system (RAAS) blockade on reducing pathological fibrosis and myocardial hypertrophy and improving cardiac function by blocking AngII receptors (27). In fact, this supports the importance of increasing the ANP level due to NEP inhibition in our study. Sacubitril and valsartan produced more effective results in the groups treated with metformin, sacubitril, and valsartan alone. Therefore, neprilysin inhibitors or ARBs are more effective when used in combination than alone.

When women who develop PCOS are not adequately treated, it is accompanied by metabolic abnormalities such as IR, hyperinsulinemia, dyslipidemia, obesity, cardiovascular disorders, chronic inflammation, and oxidative stress (28). Currently, 50–80% of women who develop PCOS have obesity, 30-35% have impaired glucose tolerance, and 8–10% have diabetes or a history of it (29). In general, women with PCOS exhibit decreased levels of HDL and increased concentrations of TG and LDL cholesterol compared to healthy women(29). LDL cholesterol differences have been observed to play a significant role in PCOS and remain a primary concern in the majority of affected women (29). In our study, we observed that the blood lipid profile of rats developing PCOS increased in parallel with weight gain. Additionally, HDL levels were shown to decrease. Experimental studies reported in the literature have also shown that rats developing PCOS exhibit weight gain and increased levels of TG, LDL, and TCOL, as well as decreased HDL levels (30). In our study, we observed a significant reduction in weight gain in the metformin, sacubitril, and sacubitril+valsartan groups, whereas the valsartan group showed a less pronounced reduction. When the blood lipid profiles of the metformin, sacubitril, valsartan, and sacubitril+valsartan groups were analysed, a significant improvement compared to the PCOS group was noted. This effect was particularly evident in the valsartan and sacubitril+valsartan groups. In a study by Nikolic *et al*. on rats with induced metabolic syndrome, sacubitril+valsartan treatment was demonstrated to reduce serum TG, TC, and LDL cholesterol levels, while increasing HDL cholesterol levels (31). In our study, the increased NEP levels in groups developing PCOS were correlated with weight gain and lipid profile, which supports our findings.

It has been suggested that the underlying mechanism of insulin resistance in PCOS is a reduction in insulin receptor autophosphorylation(32). The mechanisms through which insulin resistance exerts its effects have only recently been well defined (33). In the liver and skeletal muscle, insulin resistance increases lipolysis by accumulating unesterified fatty acids. Intrahepatic lipid accumulation activates the diacylglycerol-protein kinase C axis, which inhibits the insulin receptor and affects insulin signalling and, subsequently, gluconeogenesis. In skeletal muscle, inhibiting phosphoinositide-3 kinase and phosphorylating insulin receptor substrate 1 impair insulin signaling by changing GLUT4 expression and glucose uptake (33, 34).

In our study, we demonstrated that rats developing PCOS had impaired OGTT results, increased insulin levels, and higher HOMA-IR values compared to the control group. Immunohistochemical analysis revealed significantly reduced levels of IR, IRS1, and IRS2 in the liver, muscle, and adipose tissue compared to the control group. No improvement in IR, IRS1, and IRS2 levels was observed in the sacubitril-only groups, whereas improvement was observed in the metformin, valsartan, and sacubitril+valsartan groups.

Overexpression of NEP has been associated with the development of IR and metabolic syndrome in a study involving 318 individuals with metabolic syndrome (4). Increased levels of body mass index (BMI) and IR have been correlated with NEP activity in subjects with multiple cardiovascular risk factors. In human adipocytes, NEP protein production increases during differentiation and in the bloodstream. Therefore, NEP is associated with cardiometabolic risk in cases of IR and obesity (4). Hence, NEP activity shows a positive correlation with IR and BMI in obese patients with cardiometabolic disorders (35, 36). Additionally, NEP inhibitors have also been shown to be effective in the treatment of diabetes by increasing the circulating levels of GLP-1, which is degraded by NEP (37). In our study, we observed that NEP levels increased alongside PCOS and the accompanying IR, while NEP levels decreased and IR was reduced in the sacubitril+valsartan group.

In a study, it was demonstrated that the sacubitril+valsartan combination exhibited a therapeutic effect in rats with induced metabolic syndrome by increasing the levels of peroxisome proliferator-activated receptor (PPAR-γ), mammalian target of rapamycin complex 1 (mTORC1), and uncoupling protein 1 (UCP-1) in white adipose tissue (31). This study observed that impaired OGTT due to metabolic syndrome improved with sacubitril and valsartan combination treatment. Furthermore, the study showed that sacubitril+valsartan combination treatment normalised the elevated insulin levels associated with metabolic syndrome. Furthermore, a clinical study comparing the metabolic effects of amlodipine and sacubitril/valsartan in obese patients with hypertension reported that sacubitril/valsartan treatment improved metabolic parameters and enhanced insulin sensitivity (38). These data are consistent with, and provide support for, the findings of our study.

While only cornified epithelial cells were observed in the estrus phase in the polycystic ovary syndrome (PCOS)-treated groups (Figure 1-A), cornified epithelial cells and leukocytes were observed in the metaestrus phase in the healthy group animals (Figure 1-B)

*: Comparison versus control, #: Comparison versus PCOS group. ** and ^##^*P<*0.01, *** and ^###^*P<*0.001 indicate significance levels. Mean±SD values were used

C: control, Met: metformin, Sac: sacubitril, Val: valsartan, OGTT: Oral glucose tolerance test, PCOS: Polycystic ovary syndrome, HOMA-IR: Homeostatic model assessment - insulin resistance

A) Serum estrogen level, B) Serum testosterone level, C) Serum FSH level, D) Serum LH level. *: Comparison versus control, #: Comparison versus PCOS group. * and #*P<*0.05, ** and ^##^*P<*0.01, *** and ^###^*P<*0.001 indicate significance levels. Mean±SD values were used

C: control, Met: metformin, Sac: sacubitril, Val: valsartan, FSH: Follicle-stimulating hormone, LH: Luteinizing hormone, PCOS: Polycystic ovary syndrome

A) Plasma Neprilysin level, B) Plasma ANP level, C) Plasma BNP level, D) Plasma AngII level. *: Comparison versus control, #: Comparison versus PCOS group. * and ^#^*P<*0.05, ** and ^##^*P<*0.01, *** and ^###^*P<*0.001 indicate significance levels. Mean±SD values were used

A) Serum HDL level, B) Serum LDL level, C) Serum TCOL (total cholesterol) level, D) Serum TG (triglyceride) level. *: Comparison versus control, #: Comparison versus PCOS group. * and ^#^*P<*0.05, ** and ^##^*P<*0.01, *** and ^###^*P<*0.001 indicate significance levels. Mean±SD values were used

C: control, Met: metformin, Sac: sacubitril, Val: valsartan, LDL: Low-density lipoprotein, HDL: High-density lipoprotein, TCOL: Total cholesterol, TG: Triglyceride, PCOS: Polycystic ovary syndrome

Circle: Mesenchymal tissue, CL: Corpus Luteum, CF: Cystic Follicle, Circle: Unstained peripheral endocrine pancreatic cells, C: control, Met: metformin, GF: Graafian Follicle M: Medulla, O: Oocyte, PF: Primary Follicle, Square: Steatosis lipid accumulations, SF: Secondary Follicle, Star: Developing ovarian follicles, Sac: sacubitril, TH: Theca Cells, Val: valsartan

IR: Insulin Receptor, IRS1: Insulin Receptor Substrate 1, IRS2: Insulin Receptor Substrate 2, +: Highly Positive Cells, -: Mildly Positive Cells, C: control, Met: metformin, Sac: sacubitril, Val: valsartan

**Table 1:** Immunohistochemical staining of the tissues from different experimental groups. Positive-negative cell scoring of IR, IRS1, and IRS2 in muscle, liver, and white adipose tissue

**GROUPS**	**Liver**			**muscle**			**wat**		
	IR	IRS1	IRS2	IR	IRS1	IRS2	IR	IRS1	IRS2
**C**	3	3	3	3	3	3	3	3	3
**PCOS**	1	1	1	2	1	1	1	1	1
**MET**	3	2	3	3	3	3	2	2	2
**SAC**	2	1	1	2	1	1	1	1	1
**VAL**	3	3	3	3	2	3	1	1	3
**SAC+VAL**	3	3	2	2	3	2	2	2	3

Immunoreactivity was scored as immune negative-(0), mildly positive-(1), moderately positive-(2) and strongly positive-(3)

IR: Insulin resistance, IRS1: Insulin receptor substrate 1, IRS2: Insulin receptor substrate 2, WAT: White adipose tissue, C: Control, Met: Metformin, Sac: Sacubitril, Val: Valsartan, PCOS: Polycystic ovary syndrome

## Conclusion

The combination of sacubitril and valsartan has shown significant therapeutic benefits in PCOS patients, including a notable reduction in cystic follicles and PCOS-related insulin resistance, along with improvements in serum lipid levels. Pathological findings support these benefits. Therefore, the sacubitril and valsartan combination is considered a promising treatment option for women with PCOS, especially given its potential cardiovascular advantages, which further underscore its clinical importance.

## Declaration

We have not used any AI tools or technologies to prepare this manuscript.
